# Supplementary material for: Plasma Next Generation Sequencing and Droplet Digital-qPCR-Based Quantification of Circulating Cell-Free RNA for Noninvasive Early Detection of Cancer
Source: Cancers (Basel). 2020 Feb 4;12(2):353. doi: 10.3390/cancers12020353 (PMC7073169; doi:10.3390/cancers12020353)
Supplement: Supplementary file 1 [file cancers-12-00353-s001.pdf]

cancers

Article

Plasma Next Generation Sequencing and Droplet Digital-qPCR-Based Quantification of Circulating Cell-Free RNA for Noninvasive Early Detection of Cancer

Martin Metzenmacher, Renáta Váraljai, Balazs Hegedüs, Igor Cima, Jan Forster, Alexander Schramm, Björn Scheffler, Peter A. Horn, Christoph A. Klein, Tibor Szarvas, Hennig Reis, Nicola Bielefeld, Alexander Roesch, Clemens Aigner Volker Kunzmann, Marcel Wiesweg, Jens T. Siveke, Martin Schuler and Smiths S. Lueong

Supplementary Materials

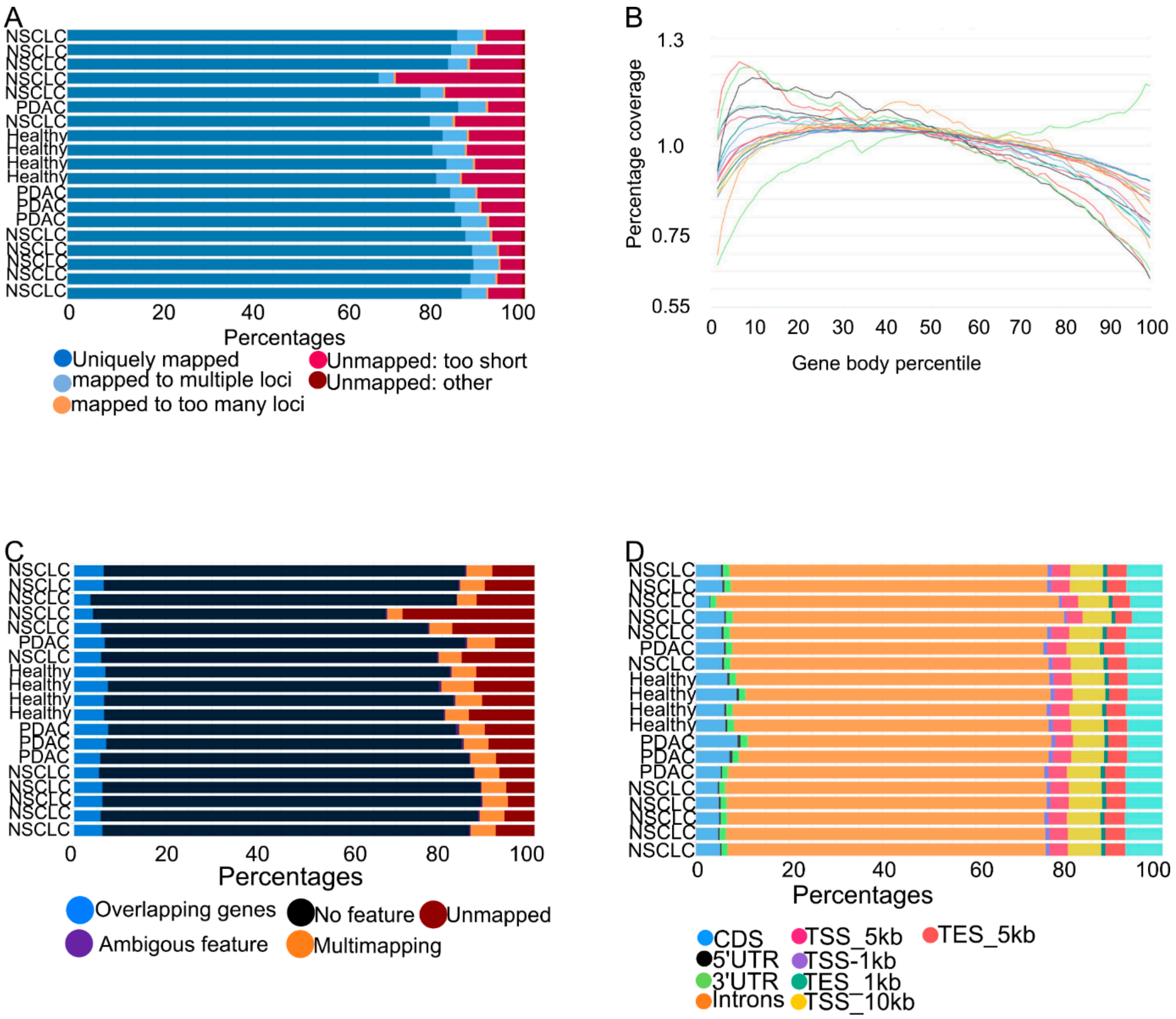

Supplementary Figure S1. Total cfRNA sequencing quality control as estimated by STAR and RSEQC. (**A**) Mapping statistics across all sequenced samples as reported by the STAR algorithm. (**B**) Gene body coverage across all samples as reported by RSSEQC algorithm. (**C**) Gene count statistics as reported by the STAR algorithm across all sequenced samples. (**D**) Read distribution across all sequenced samples as reported by the RSEQC algorithm.

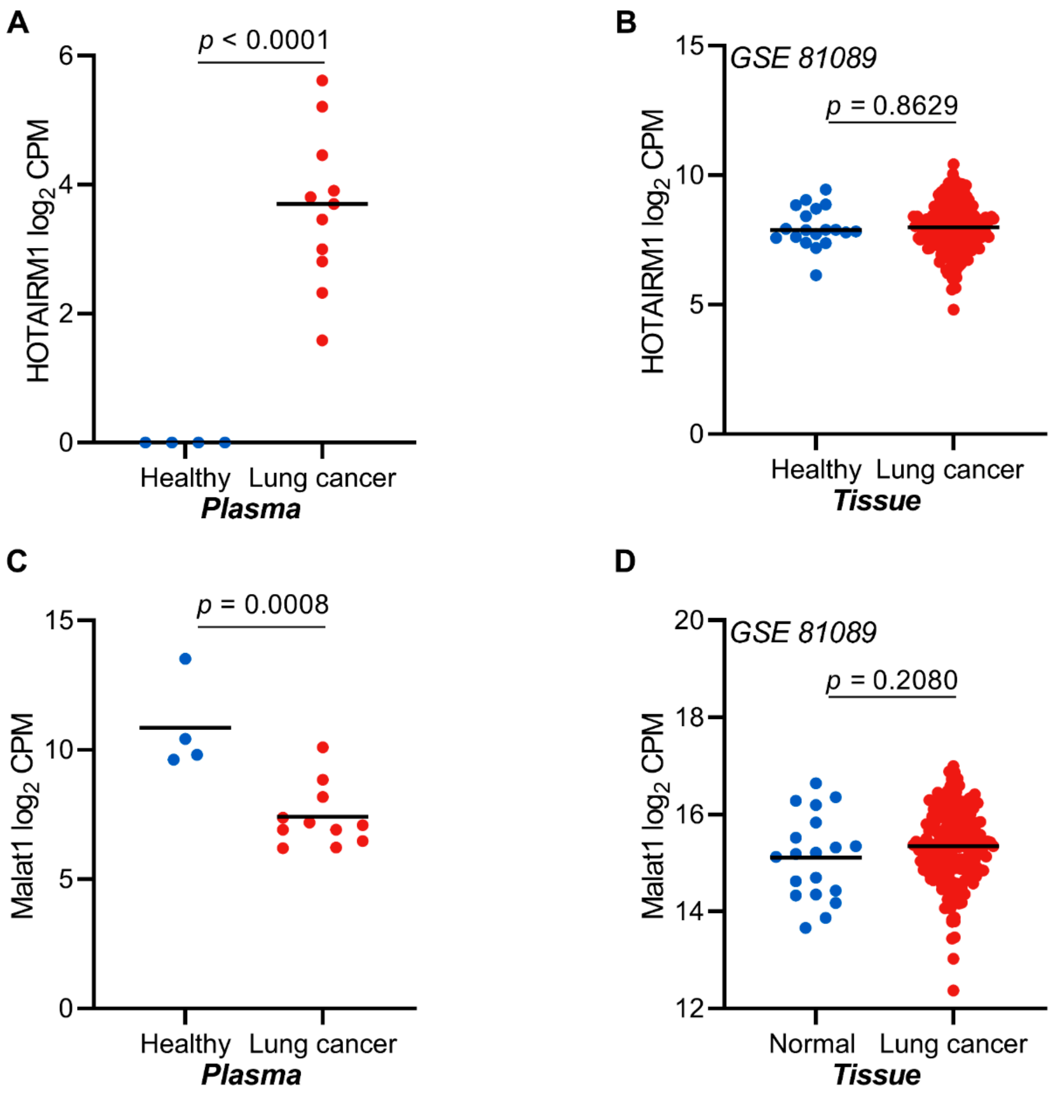

Supplementary Figure S2. Tissue expression of HOTAIRM1 and MALAT1 in Lung cancer tissue and non-tumor lung tissue. (**A**) Plasma expression of *HOTAIRM1* as profiled by total cfRNA sequencing in healthy donors ($n = 4$) and NSCLC ($n = 11$). (**B**) Tissue expression of *HOTAIRM1* as profiled by RNA sequencing (GSE 81089) in healthy donors ($n = 19$) and NSCLC ($n = 199$). (**C**) Plasma expression of *MALAT1* as profiled by total cfRNA sequencing in healthy donors ($n = 4$) and NSCLC ($n = 11$). (**D**) Tissue expression of *MALAT1* as profiled by RNA sequencing (GSE 81089) in healthy donors ($n = 19$) and NSCLC ($n = 199$).

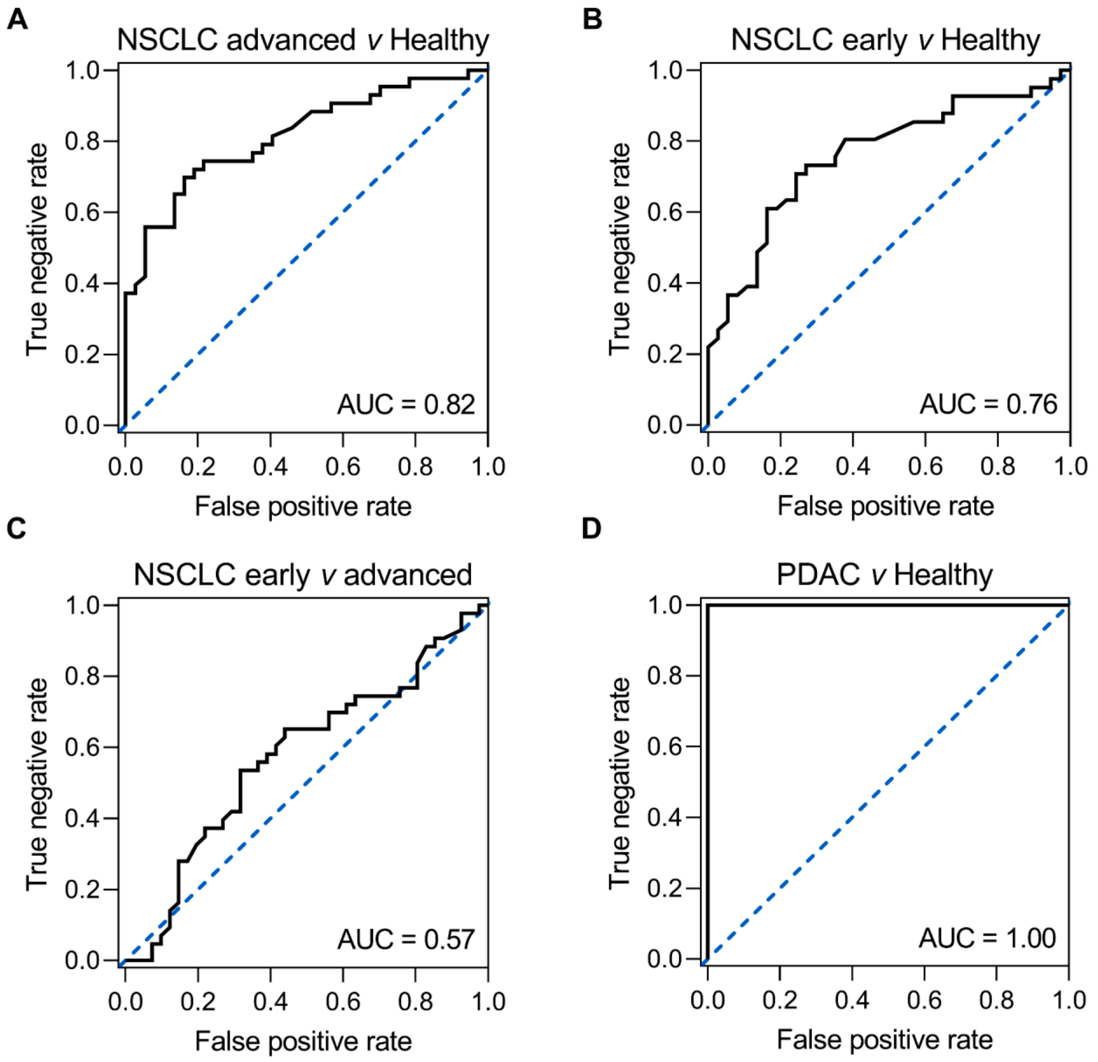

Supplementary Figure S3. Diagnostic performance of *POU6F2-AS2* in NSCLC and serum total cfRNA in PDAC. Receiver operating characteristics (ROC) curve of *POU6F2-AS2* expression (**A**) in healthy donors' (*n* = 37) and advanced stage NSCLC (*n* = 45) plasma samples (**B**) in healthy donors' (*n* = 37) and early stage NSCLC (*n* = 39) plasma samples (**C**) in advanced stage NSCLC (*n* = 45) and early stage NSCLC (*n* = 39) plasma samples and (**D**) in healthy donors' (*n* = 23) and PDAC (*n* = 20) serum samples. Area under the curve (AUC) value indicates the diagnostic accuracy. Dashed line indicates the line of identity.

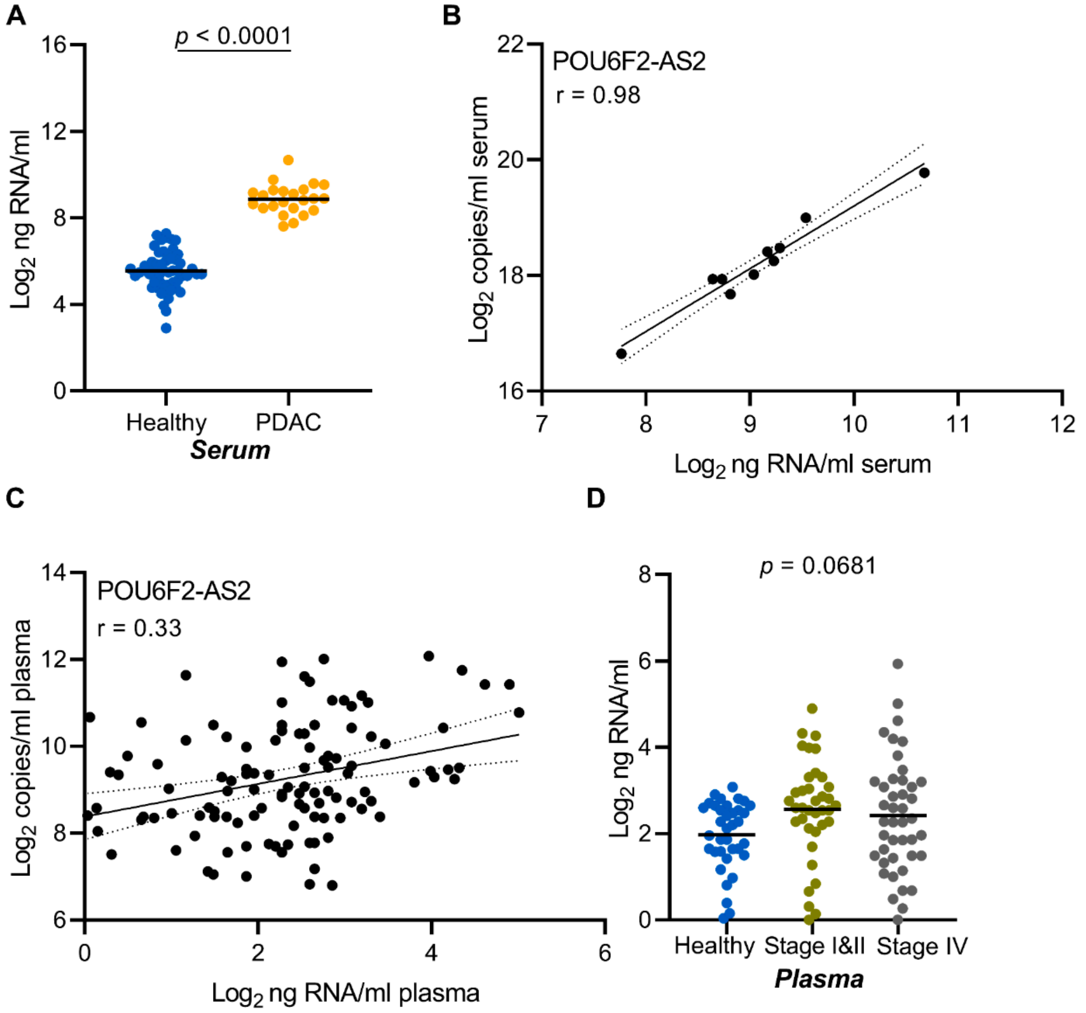

Supplementary Figure S4: Association between total cfRNA concentration and *POU6F2-AS2* copies in plasma and serum. (**A**) Total serum cfRNA amount in healthy donors (*n* = 22), PDAC patients (*n* = 20) serum samples. Student's t-test *p* value is reported. (**B**) Correlation scatter plot of total serum cfRNA amount and *POU6F2-AS2* cfRNA copies in 1 ml serum samples (*n* = 10) of PDAC patients. In A, C scatter dot-plots, the line indicates the mean. (**C**) Correlation scatter plot of total plasma cfRNA amount and POU6F2-AS2 cfRNA copies in 1 mL plasma samples (*n* = 84) of NSCLC patients (**D**) Total plasma cfRNA amount in healthy donors (*n* = 37), early stage NSCLC (*n* = 39) and late stage NSCLC (*n* = 45). Significance was tested in 1-way ANOVA.

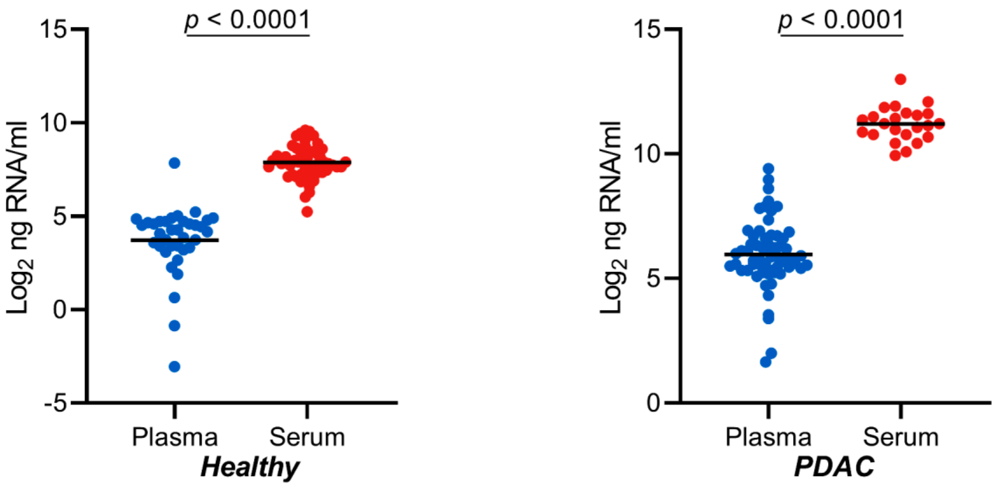

Supplementary Figure S5. Total cfRNA yield is higher in serum compared with plasma. (**A**) total cfRNA yield per ml of serum (n = 52) and plasma (n = 36) in healthy blood donors. (**B**) Total cfRNA yield in PDAC plasma (n = 62) and serum (n= 23) samples. In A-B scatter dot-plots, the line indicates the mean; Student's t-test p values are indicated.

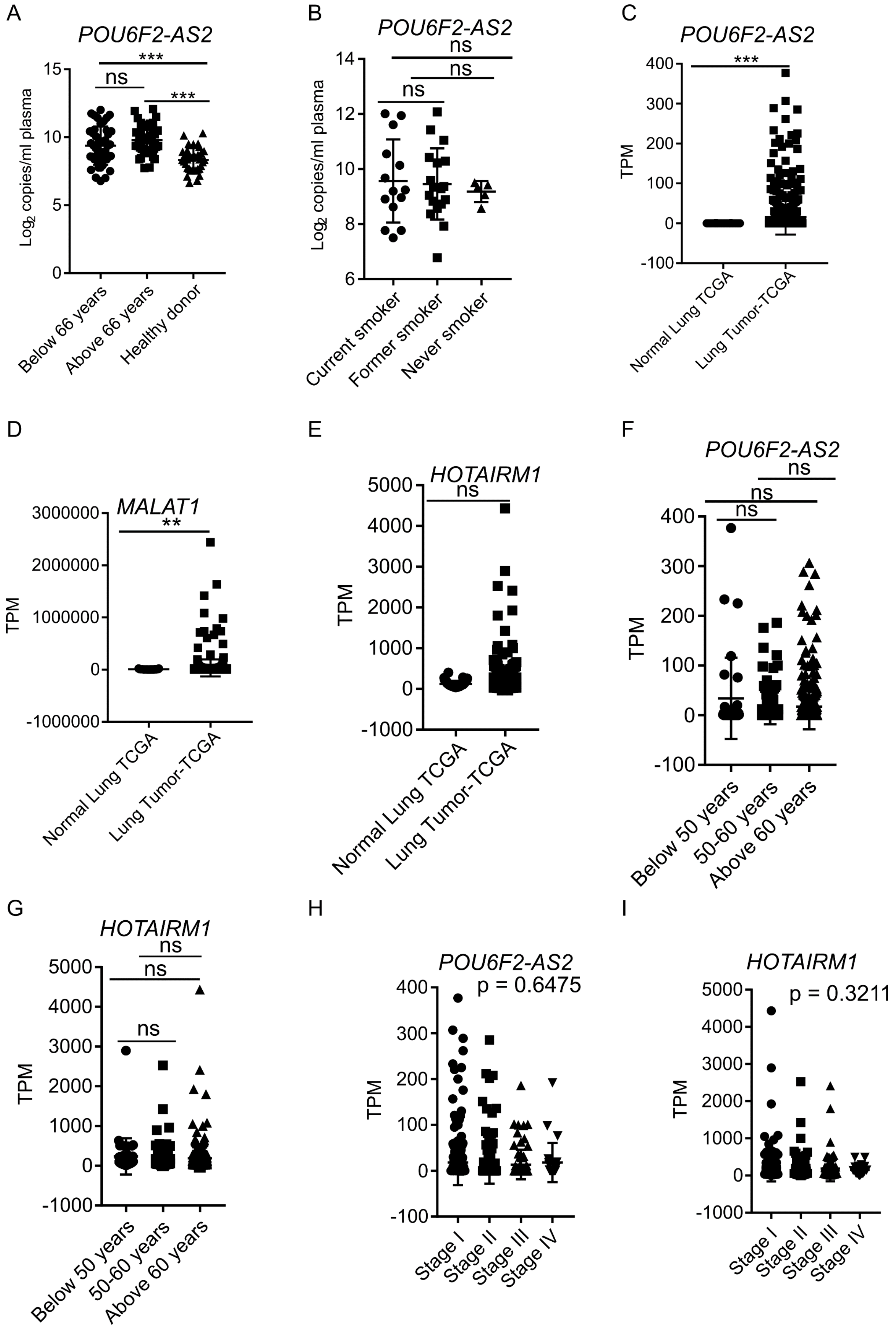

Supplementary Figure S6. Transcript expression data for *HOTAIRM1*, *MALAT1* and *POU6F2-AS2* in cfRNA and the TCGA data set. (**A**) Expression of *POU6F2-AS2* in different age groups in our patient

population as measured by RT-ddPCR. (**B**) Expression of *POU6F2-AS2* in smokers, former smokers and never smokers in our patient population as measured by RT-ddPCR. (**C**) Expression of *POU6F2-AS2* in tumor tissue and non-tumor tissue in the TCGA data set. (**D**) Expression of *MALAT1* in tumor tissue and non-tumor tissue in the TCGA data set. (**E**) Expression of *HOTAIRM1* in tumor tissue and non-tumor tissue in the TCGA data set. (**F**) Expression of *POU6F2-AS2* in different age groups of the patient population in the TCGA data set. (**G**) Expression of *HOTAIRM1* in different age groups of the patient population in the TCGA data set. (**H**) Expression of *POU6F2-AS2* in different disease stages of the patient population in the TCGA data set. **(I**) Expression of *HOTAIRM1* in different disease stages of the patient population in the TCGA data set.
